# Author Correction: Fast analysis and engineering of protein function by microbe-independent deep assembly and screening

**DOI:** 10.1038/s44320-026-00233-6

**Published:** 2026-07-16

**Authors:** Yan Wu, Pengli Wang, Lan Xiang Liu, Daesun Song, Qin Qin, Chao Gao, Matt Hageman, Thomas A Kirkland, Yichi Su, Michael Z Lin

**Affiliations:** 1 Department of Bioengineering, Stanford University, Stanford, CA USA; 2 Department of Chemical Engineering, Stanford University, Stanford, CA USA; 3 Department of Neurobiology, Stanford University, Stanford, CA USA; 4 Institute for Translational Brain Research, State Key Laboratory of Medical Neurobiology, MOE Frontiers Center for Brain Science, Fudan University, Shanghai, China; 5 Promega Corporation, San Luis Obispo, CA USA; 6 Promega Corporation, Madison, WI USA; 7 Department of Nuclear Medicine, Zhongshan Hospital, Shanghai, China; 8 Department of Pediatrics, Stanford University, Stanford, CA USA; 9 Department of Chemical and Systems Biology, Stanford University, Stanford, CA USA; 10 Present Address: Department of Antibody Engineering, Genentech Inc., South San Francisco, CA USA

## Abstract

**Figure:**
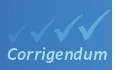

**Correction to:** *Molecular Systems Biology* (2026) 22:1003–1034. https://doi.org/10.1038/s44320-026-00210-z | Published online 23 April 2026

**The Figure 5 legend is corrected**

The Figure 5 legend is corrected from:

**Figure 5. MIDAS-MM concept and application to rapid deep enzyme characterization and improvement.** (**A**) Organization of construct for assessing NanoLuc variant activity relative to FLuc. (**B**) Sequence and structure of NanoLuc luciferase bound to substrate analog 3-methoxy-furimazine (PDB:7SNT). The side chains of the 25 residues selected for saturation mutagenesis are shown as white sticks; the 23 that are not Gly or Ala are labeled. (**C**) Relative catalytic activity of all 20 NanoLuc variants at each of the 25 residues on 5 μM of Fz, FFz, or CFz-9 after generation and expression by MIDAS-MM. Mean normalized score below the maps indicates the mutational tolerance at each active-site position. Asterisks, sites showing tolerance selected for later analysis. Daggers, sites showing intolerance selected for later analysis. (**D**) Relative catalytic activity of all 20 NanoLuc variants expressed by MIDAS-MM or after plasmid cloning and transfection. left: WT vs D108N in amplicon: *p* = 0.0008, in plasmid: *p* = 0.0006; middle: WT vs D108N in amplicon: *p* = 0.0042, in plasmid: *p* = 0.0004, WT vs D108S in amplicon: *p* = 0.0079, in plasmid: *p* = 0.0003; left: WT vs D108N in amplicon: *p* = 0.0042, in plasmid: *p* = 0.0001, *p < 0.05; **p < 0.01; ***p < 0.001; ****p < 0.0001, by unpaired two-tailed Student’s t-test compared to the saline control. Error bars, SDs. Three technical replicates. (**E**) Models of NanoLuc with tolerance scores at each active-site residue, coupled with Fz (left), FFz (middle) and CFz-9 (right), respectively. Selected tolerant and intolerant sites are indicated. Source data are available online for this figure.

**Corrected to:**

**Figure 5. MIDAS-MM concept and application to rapid deep enzyme characterization and improvement.** (**A**) Organization of the construct for assessing NanoLuc variant activity relative to FLuc and sequence of NanoLuc with active-site positions in red. (**B**) Structure of NanoLuc luciferase bound to substrate analog 3-methoxy-furimazine (PDB:7SNT). The side chains of the 25 residues selected for saturation mutagenesis are shown as white sticks; the 23 that are not Gly or Ala are labeled. (**C**) Relative catalytic activity of all 20 NanoLuc variants at each of the 25 residues on 5 μM of Fz, FFz, or CFz9 after generation and expression by MIDAS-MM. Mean normalized score below the maps indicates the mutational tolerance at each active-site position. Asterisks, sites showing tolerance, selected for later analysis. Daggers, sites showing intolerance selected for later analysis. (**D**) Relative catalytic activity of selected NanoLuc variants expressed by MIDAS-MM or after plasmid cloning and transfection. *p ≤ 0.05; **p ≤ 0.01; ***p ≤ 0.001; ****p ≤ 0.0001, by unpaired two-tailed Student’s *t*-test compared to the saline control. Error bars, SDs. Three technical replicates. Precise *p* values are: Fz amplicon WT vs D108N, *p* = 0.0008; Fz plasmid WT vs D108N in plasmid, *p* = 0.0006; FFz amplicon WT vs D108N, *p* = 0.0042; FFz amplicon WT vs D108S, *p* = 0.0079; FFz plasmid WT vs D108N, *p* = 0.0004; FFz plasmid WT vs D108S, *p* = 0.0003; CFz9 amplicon WT vs D108N, *p* = 0.0042; CFz9 plasmid WT vs D108N, *p* = 0.0001. (**E**) Models of NanoLuc with tolerance scores at each active-site residue with Fz, FFz, or CFz9. Selected tolerant and intolerant sites are indicated. Source data are available online for this figure.

