# Supplementary material for: Fast analysis and engineering of protein function by microbe-independent deep assembly and screening
Source: Mol Syst Biol. 2026 Apr 23;22(6):1003–34. doi: 10.1038/s44320-026-00210-z (PMC13230610; doi:10.1038/s44320-026-00210-z)
Supplement: Supplementary file 2 — Table EV1 [file 44320_2026_210_MOESM2_ESM.pdf]

Name	Protein sequence
Color code	Ig Kappa leader sequence; mScarlet-I; OpuBC(30-106); SmBiT(ΔC3); LgBiT; OpuBC(107-305); PDGFRβ TM domain; linker
ACh-NeuBI0.5	-20 METDTLLLWVLLLWVPGSTGD<br>1 MVSKGEAVIKEFMRFKVHMEGSMNGHEFEIEGEGEGRPYEGTQTAKLKVT<br>51 KGGPLPFSWDILSPQFMYGSRAFIKHPADIPDYYKQSFPEGFKWERVMNF<br>101 EDGGAVTVTQDTSLEDGTLIYKVKLRGTNFPPDGPVMQKKTMGWEASTER<br>151 LYPEDGVLKGDIKMALRLKDGGRYLADFKTTYKAKKPVQMPGAYNVDRKL<br>201 DITSHNEDYTVVEQYERSEGRHSTGGGRSANDTVVVGSIIFTEGIIVANM<br>251 VAEMIEAHTDLKVVRKLNLGGVNVNFEAIKRGGANNGIDIYVEYTGHGLV<br>301 DILGFPFVTGYRLFEGGSGGSFTLEDFVGDWEQTAAYNLDQVLEQGGVSS<br>351 LLQNLAVSVTPIQRIVRSGENALKIDIHVIIPYEGLSADQMAQIEEVFKV<br>401 VYPVDDHHFKVILPYGTLVIDGVTPNMLNYFGRPYEGIAVFDGKKITVTG<br>451 TLWNGNKIIDERLITPDGSMLFRVTINSATTDPEGAYETVKKEYKRKWNI<br>501 VWLKPLGFNNTYTLTVKDELAKQYNLKTFSDLAKISDKLILGATMFFLEG<br>551 PDGYPGLQKLYNFKFKHTKSMDMGIRYTAIDNNEVQVIDAWATDGLLVSH<br>601 KLKILEDDKAFFPPYYAAPIIRQDVLDKHPELKDVLNKLANQISLEEMQK<br>651 LNYKVDGEGQDPAKVAKEFLKEKGLILQVDEQKLISEEDLNAVGQDTQEV<br>701 IVVPHSLPFKVVVISAILALVVLTIISLIILIMLWQKKPR
ACh-NeuBI1b	-20 METDTLLLWVLLLWVPGSTGD<br>1 MVSKGEAVIKEFMRFKVHMEGSMNGHEFEIEGEGEGRPYEGTQTAKLKVT<br>51 KGGPLPFSWDILSPQFMYGSRAFIKHPADIPDYYKQSFPEGFKWERVMNF<br>101 EDGGAVTVTQDTSLEDGTLIYKVKLRGTNFPPDGPVMQKKTMGWEASTER<br>151 LYPEDGVLKGDIKMALRLKDGGRYLADFKTTYKAKKPVQMPGAYNVDRKL<br>201 DITSHNEDYTVVEQYERSEGRHSTGGGRSANDTVVVGSIIFTEGIIVANM<br>251 VAEMIEAHTDLKVVRKLNLGGVNVNFEAIKRGGANNGIDIYVEYTGHGLV<br>301 DILGFPFVTGYRLFEGGSGGSFTLEDFVGDWEQTAAYNLDQVLEQGGVSS<br>351 LLQNLAVSVTPIQRIVRSGENALKIDIHVIIPYEGLSADQMAQIEEVFKV<br>401 VYPVDDHHFKVILPYGTLVIDGVTPNMLNYFGRPYEGIAVFDGKKITVTG<br>451 TLWNGNKIIDERLITPDGSMLFRVTINSATTDPEGAYETVKKEYKRKWNI<br>501 VWLKPLGFNNTYTLTVKDELAKQYNLKTFSDLAKISDKLILGATMFFLEQ<br>551 PDGYPGLQKAYNFKFKHTKSMDMGIRYTAIDNNEVQVIDAWATDGLLVSH<br>601 KLKILEDDKEFFPPYYAAPIIRQDVLDKHPELKDVLNKLANQISLEEMQK<br>651 LNYKVDGEGQDPAKVAKEFLKEKGLILQVDEQKLISEEDLNAVGQDTQEV<br>701 IVVPHSLPFKVVVISAILALVVLTIISLIILIMLWQKKPR
ACh-NeuBI1c	-20 METDTLLLWVLLLWVPGSTGD<br>1 MVSKGEAVIKEFMRFKVHMEGSMNGHEFEIEGEGEGRPYEGTQTAKLKVT<br>51 KGGPLPFSWDILSPQFMYGSRAFIKHPADIPDYYKQSFPEGFKWERVMNF<br>101 EDGGAVTVTQDTSLEDGTLIYKVKLRGTNFPPDGPVMQKKTMGWEASTER<br>151 LYPEDGVLKGDIKMALRLKDGGRYLADFKTTYKAKKPVQMPGAYNVDRKL<br>201 DITSHNEDYTVVEQYERSEGRHSTGGGRSANDTVVVGSIIFTEGIIVANM<br>251 VAEMIEAHTDLKVVRKLNLGGVNVNFEAIKRGGANNGIDIYVEYTGHGLV<br>301 DILGFPFVTGYRLFEGGSGGSFTLEDFVGDWEQTAAYNLDQVLEQGGVSS<br>351 LLQNLAVSVTPIQRIVRSGENALKIDIHVIIPYEGLSADQMAQIEEVFKV<br>401 VYPVDDHHFKVILPYGTLVIDGVTPNMLNYFGRPYEGIAVFDGKKITVTG<br>451 TLWNGNKIIDERLITPDGSMLFRVTINSATTDPEGAYETVKKEYKRKWNI<br>501 VWLKPLGFNNTYTLTVKDELAKQYNLKTFSDLAKISDKLILGATMFFLEQ<br>551 PDGYPGLQKEYNFKFKHTKSMDMGIRYTAIDNNEVQVIDAWATDGLLVSH<br>601 KLKILEDDKDFFPPYYAAPIIRQDVLDKHPELKDVLNKLANQISLEEMQK<br>651 LNYKVDGEGQDPAKVAKEFLKEKGLILQVDEQKLISEEDLNAVGQDTQEV<br>701 IVVPHSLPFKVVVISAILALVVLTIISLIILIMLWQKKPR

Table EV1. ACh-NeuBI protein sequences. Mutations introduced in ACh-NeuBI1b and ACh-NeuBI1c are underlined.
