# Supplementary figures and images for: Fast analysis and engineering of protein function by microbe-independent deep assembly and screening
Source: Mol Syst Biol. 2026 Apr 23;22(6):1003–34. doi: 10.1038/s44320-026-00210-z (PMC13230610; doi:10.1038/s44320-026-00210-z)

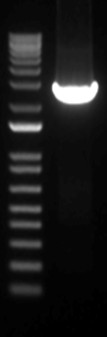

Supplement: Supplementary file 3 — Source data Fig. 2 [file 44320_2026_210_MOESM3_ESM.zip › 2b/gel image 2b.jpg]

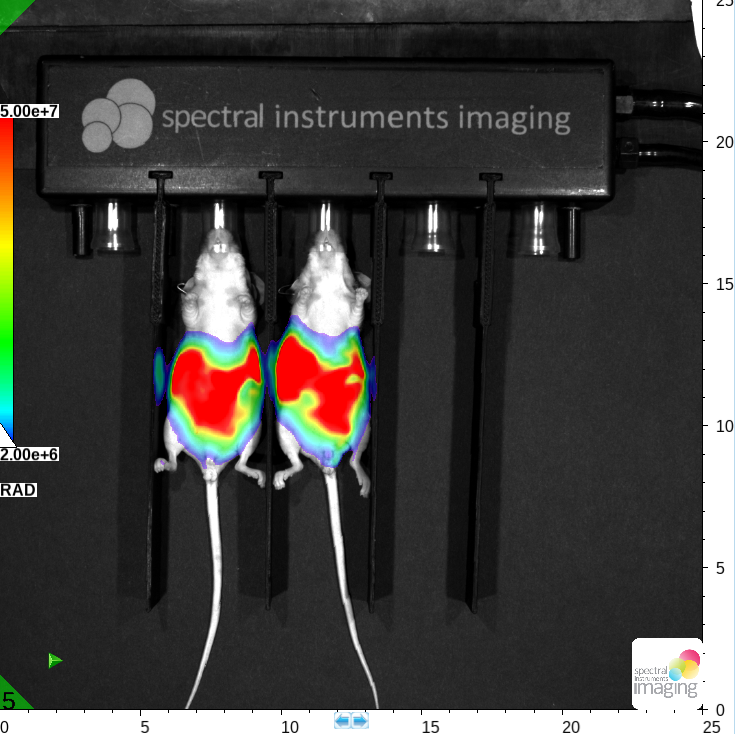

Supplement: Supplementary file 5 — Source data Fig. 4 [file 44320_2026_210_MOESM5_ESM.zip › Fig 4_YW479_hydrodynamic transfection representative images/20240418_ACh_190_post.png]

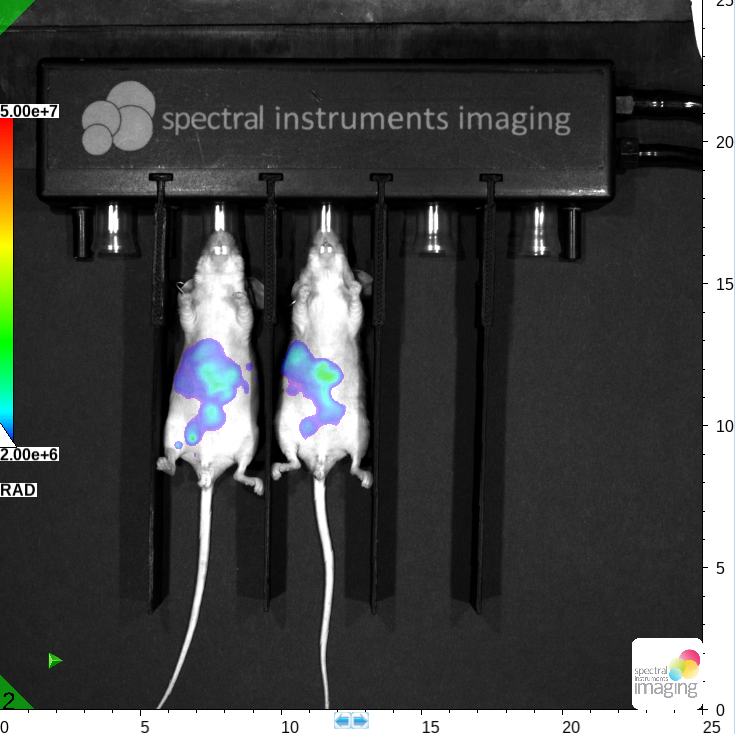

Supplement: Supplementary file 5 — Source data Fig. 4 [file 44320_2026_210_MOESM5_ESM.zip › Fig 4_YW479_hydrodynamic transfection representative images/20240418_ACh_190_pre.png]

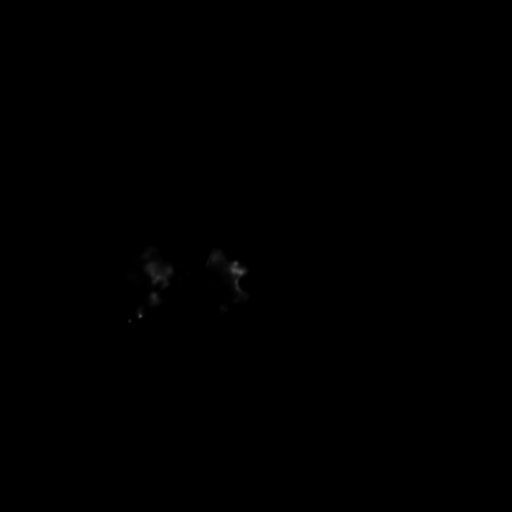

Supplement: Supplementary file 5 — Source data Fig. 4 [file 44320_2026_210_MOESM5_ESM.zip › Fig 4_YW479_hydrodynamic transfection representative images/ACh_190,191_pre-2_30s, bin4_2__lumin.png]

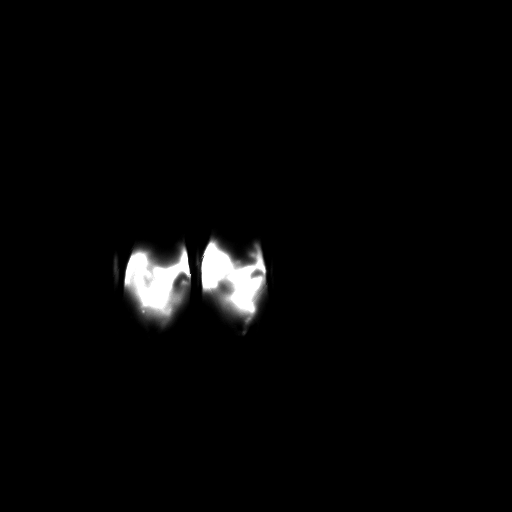

Supplement: Supplementary file 5 — Source data Fig. 4 [file 44320_2026_210_MOESM5_ESM.zip › Fig 4_YW479_hydrodynamic transfection representative images/ACh_190,191_post-2_10s,bin4_5__lumin.png]

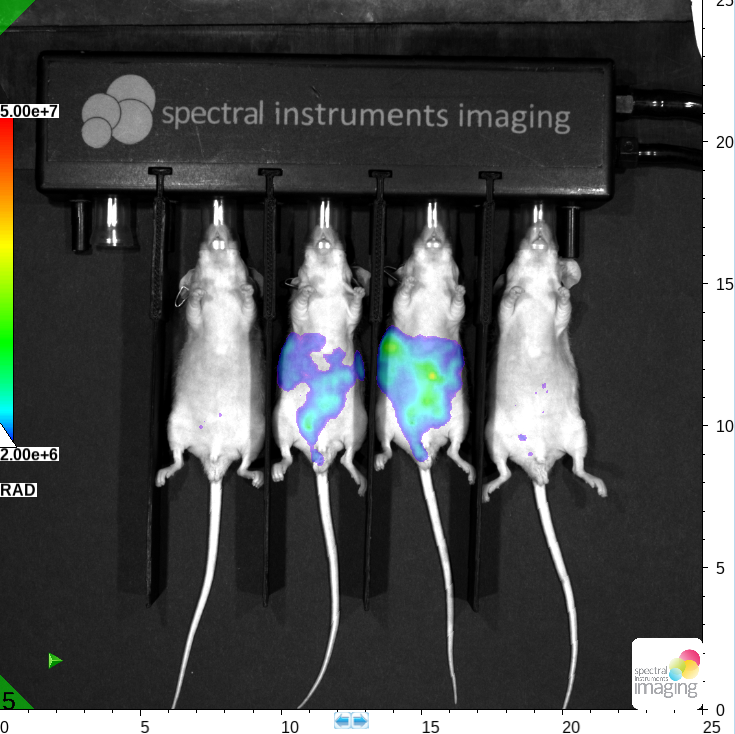

Supplement: Supplementary file 5 — Source data Fig. 4 [file 44320_2026_210_MOESM5_ESM.zip › Fig 4_YW479_hydrodynamic transfection representative images/20240418_saline_178_pre.png]

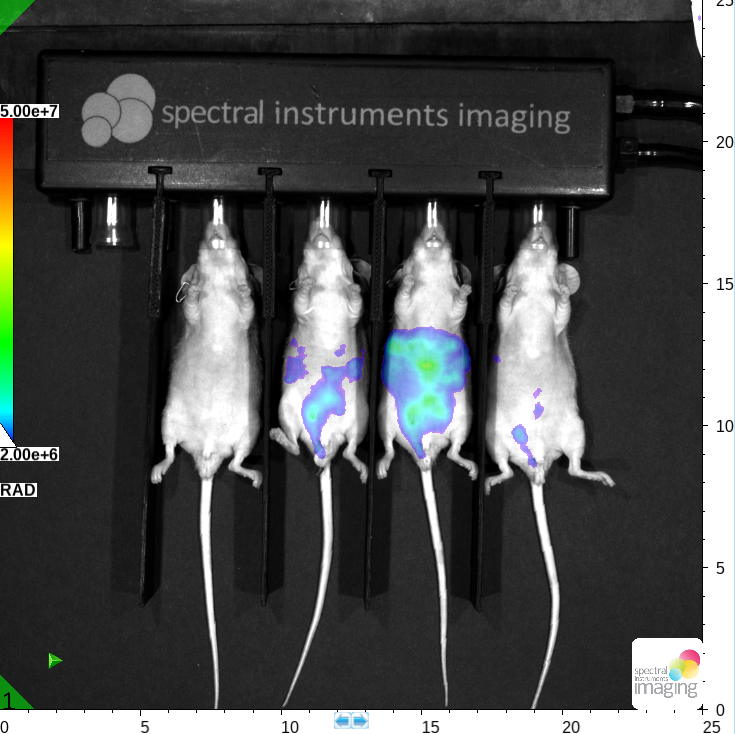

Supplement: Supplementary file 5 — Source data Fig. 4 [file 44320_2026_210_MOESM5_ESM.zip › Fig 4_YW479_hydrodynamic transfection representative images/20240418_saline_178_post.png]

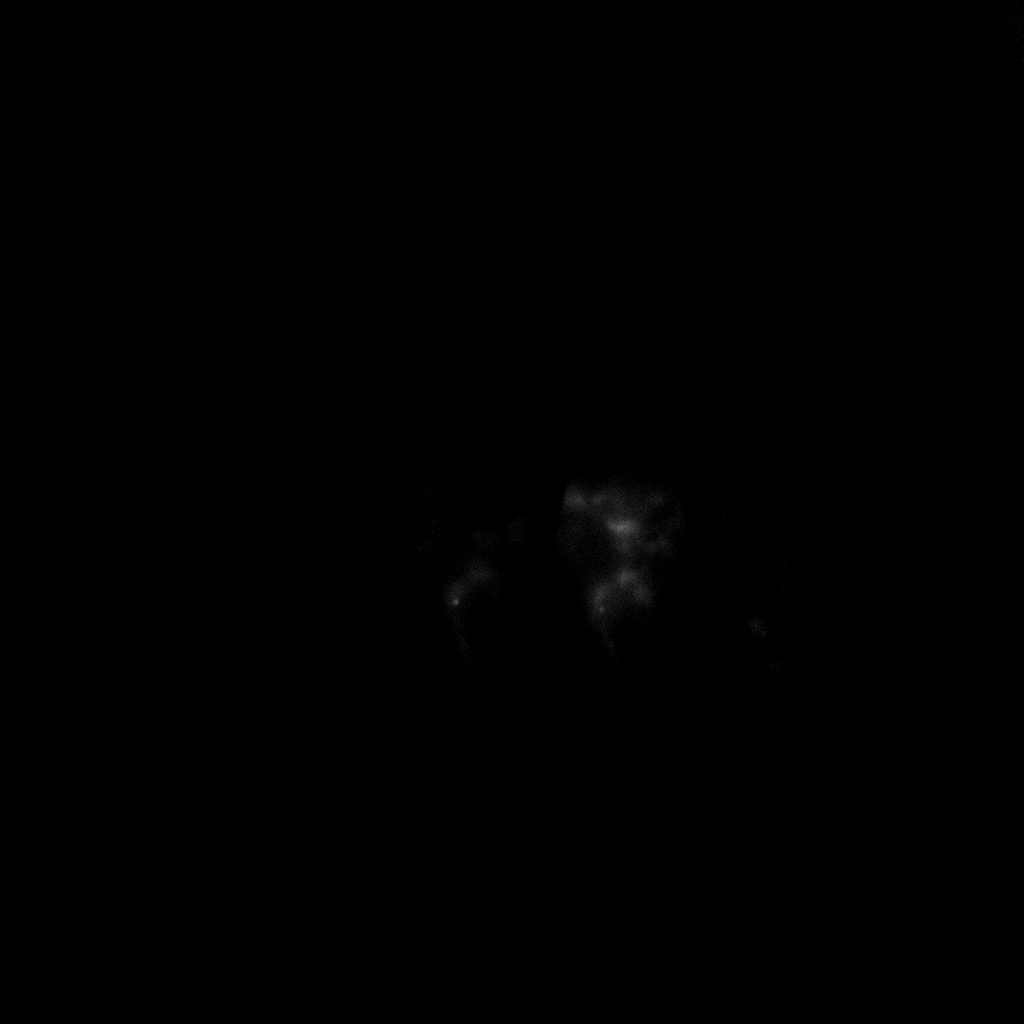

Supplement: Supplementary file 5 — Source data Fig. 4 [file 44320_2026_210_MOESM5_ESM.zip › Fig 4_YW479_hydrodynamic transfection representative images/Saline_175,177-179_post-1_5s,bin2_1__lumin.png]

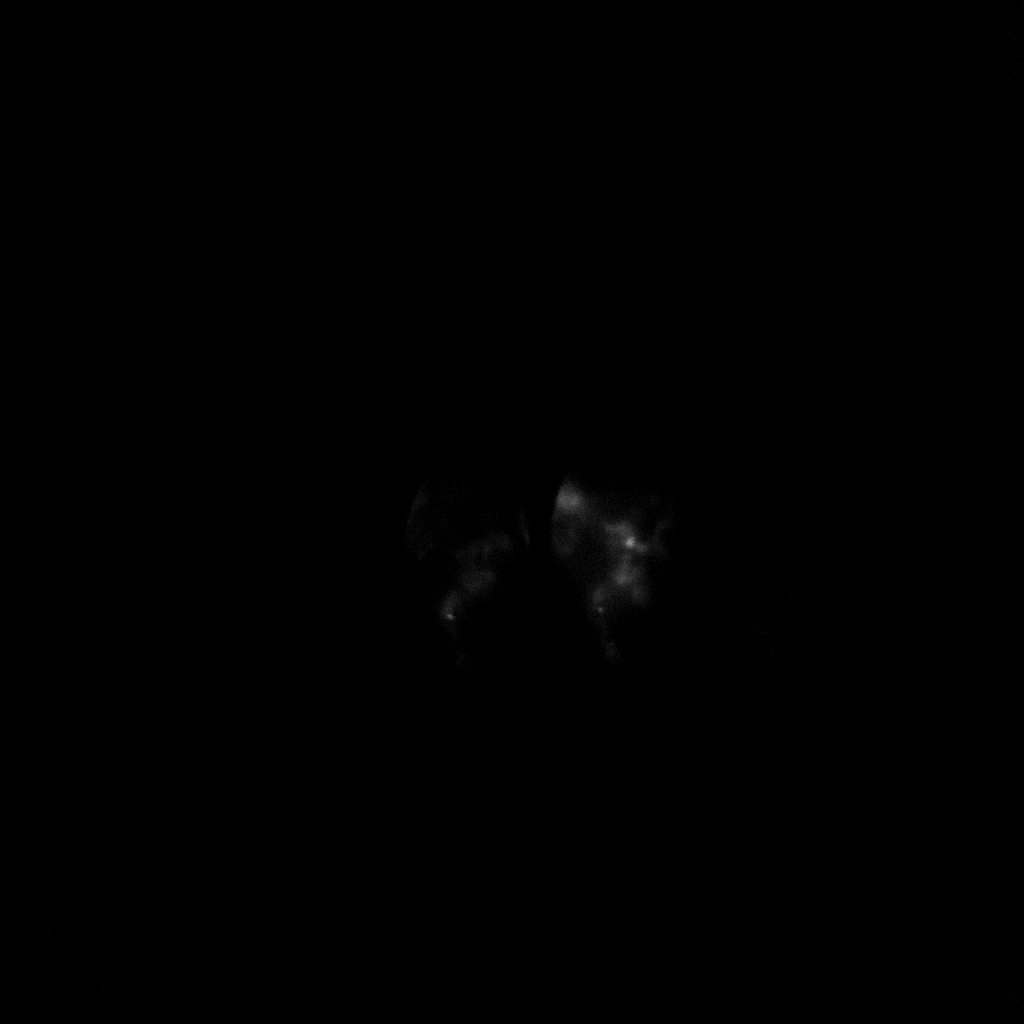

Supplement: Supplementary file 5 — Source data Fig. 4 [file 44320_2026_210_MOESM5_ESM.zip › Fig 4_YW479_hydrodynamic transfection representative images/Saline_175,177-179_pre-2_5s,bin2_5__lumin.png]

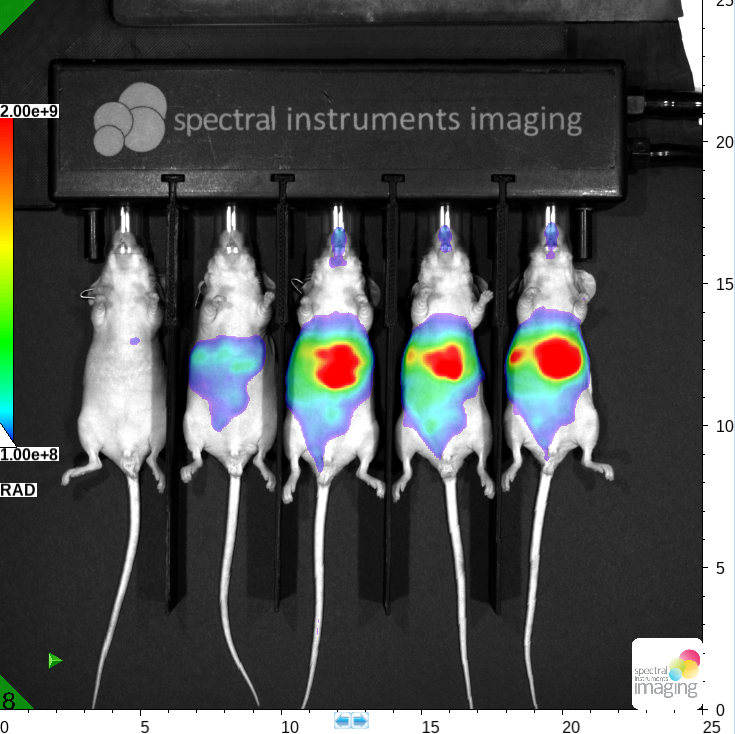

Supplement: Supplementary file 5 — Source data Fig. 4 [file 44320_2026_210_MOESM5_ESM.zip › Fig 4_YW637_hydrodynamic transfection representative images/YW637_ACh_pre (ROI4).png]

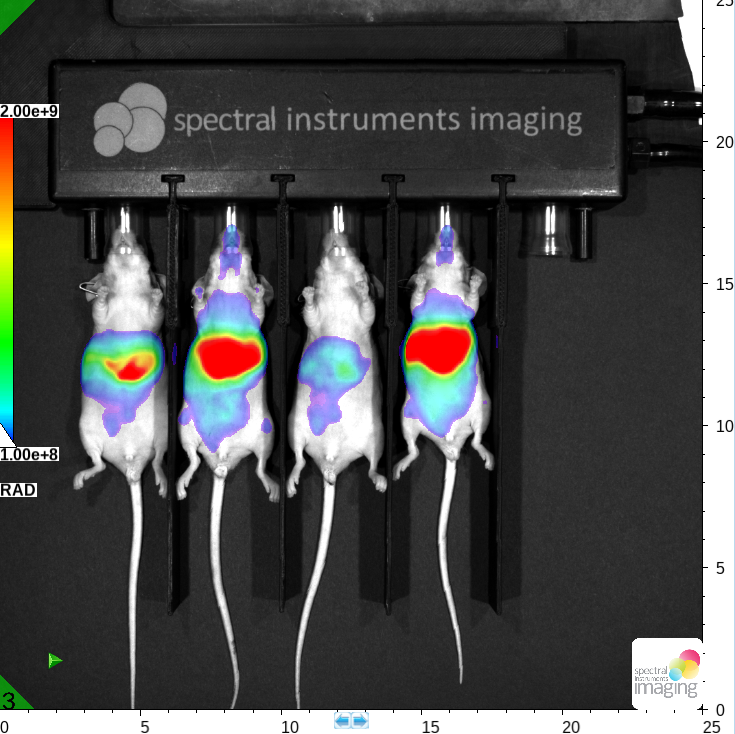

Supplement: Supplementary file 5 — Source data Fig. 4 [file 44320_2026_210_MOESM5_ESM.zip › Fig 4_YW637_hydrodynamic transfection representative images/YW637_Saline_pre (ROI1).png]

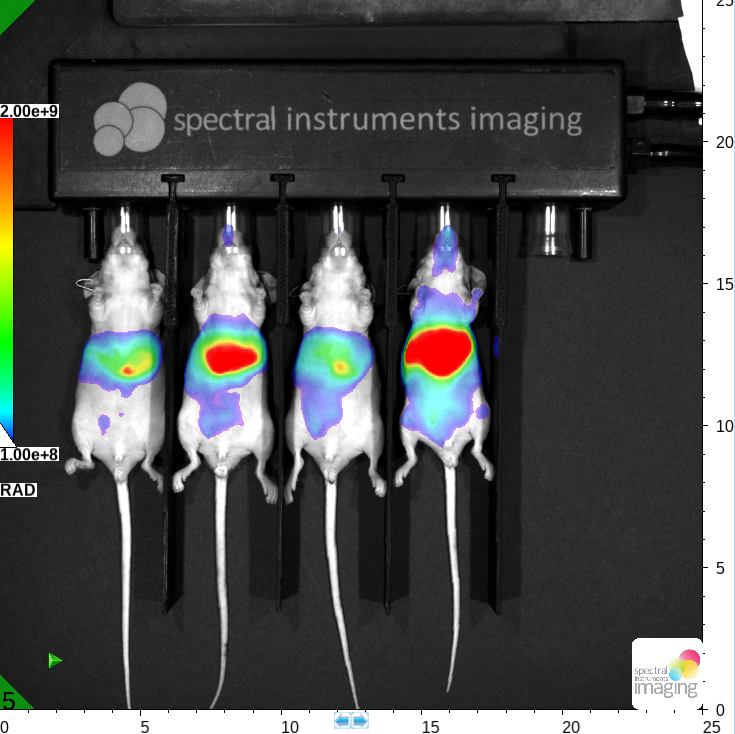

Supplement: Supplementary file 5 — Source data Fig. 4 [file 44320_2026_210_MOESM5_ESM.zip › Fig 4_YW637_hydrodynamic transfection representative images/YW637_Saline_post (ROI1).png]

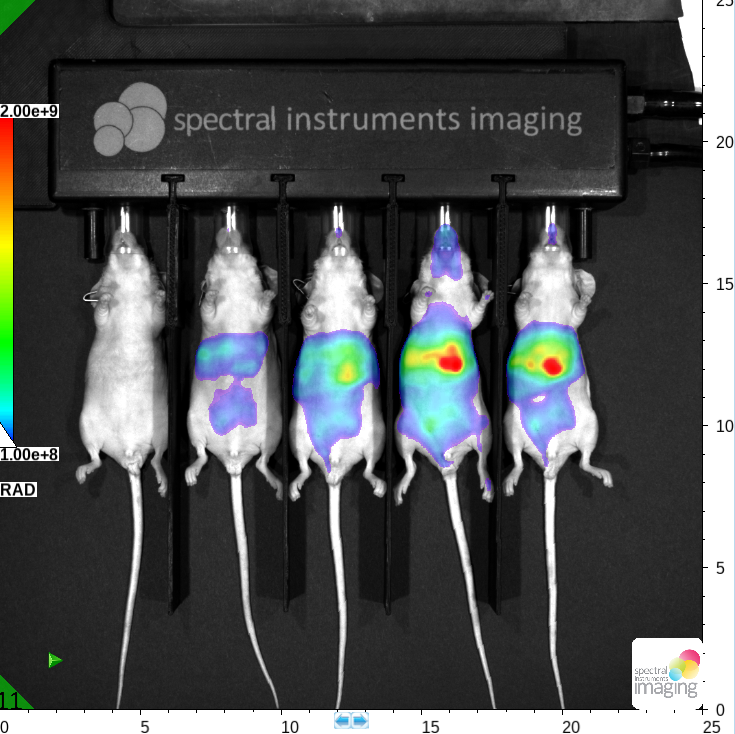

Supplement: Supplementary file 5 — Source data Fig. 4 [file 44320_2026_210_MOESM5_ESM.zip › Fig 4_YW637_hydrodynamic transfection representative images/YW637_ACh_post (ROI4).png]

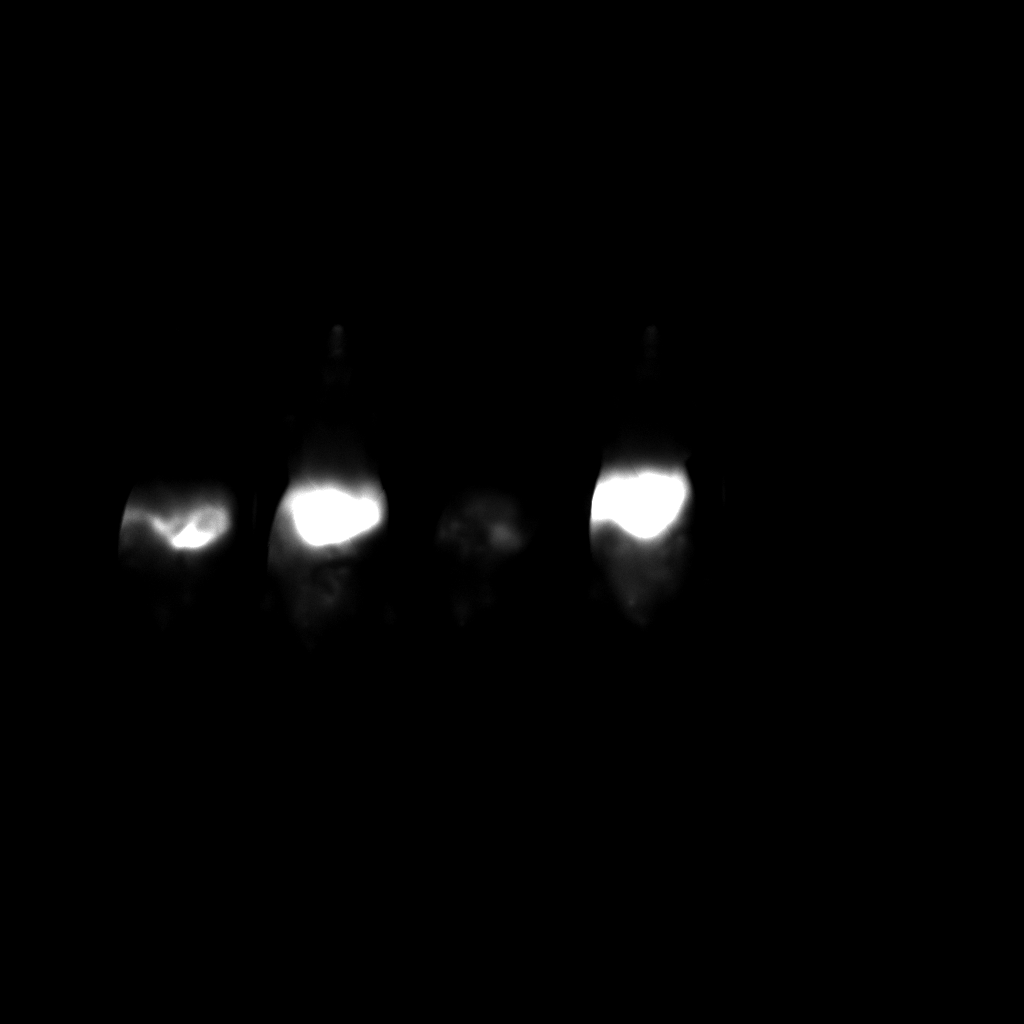

Supplement: Supplementary file 5 — Source data Fig. 4 [file 44320_2026_210_MOESM5_ESM.zip › Fig 4_YW637_hydrodynamic transfection representative images/Saline_180-184_pre-2_3__lumin.png]

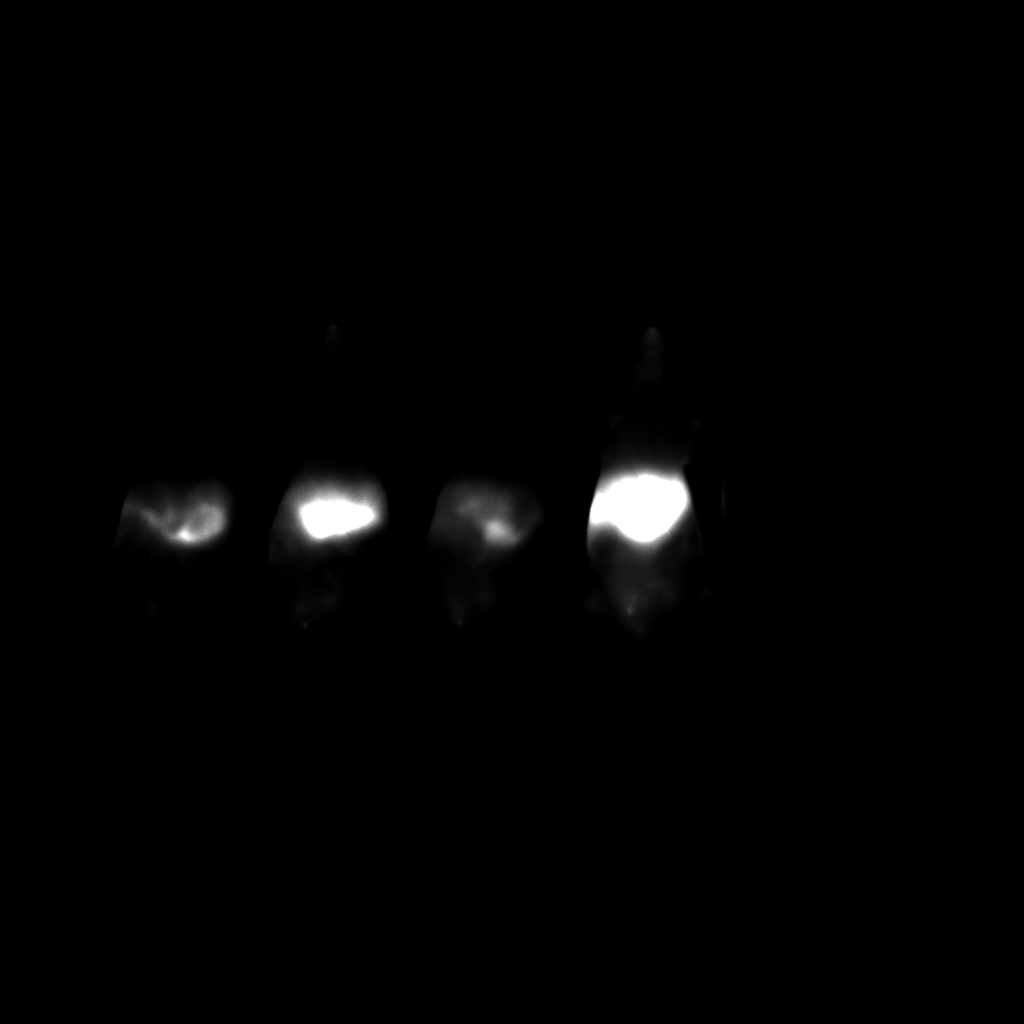

Supplement: Supplementary file 5 — Source data Fig. 4 [file 44320_2026_210_MOESM5_ESM.zip › Fig 4_YW637_hydrodynamic transfection representative images/Saline_180-184_post-1_5__lumin.png]

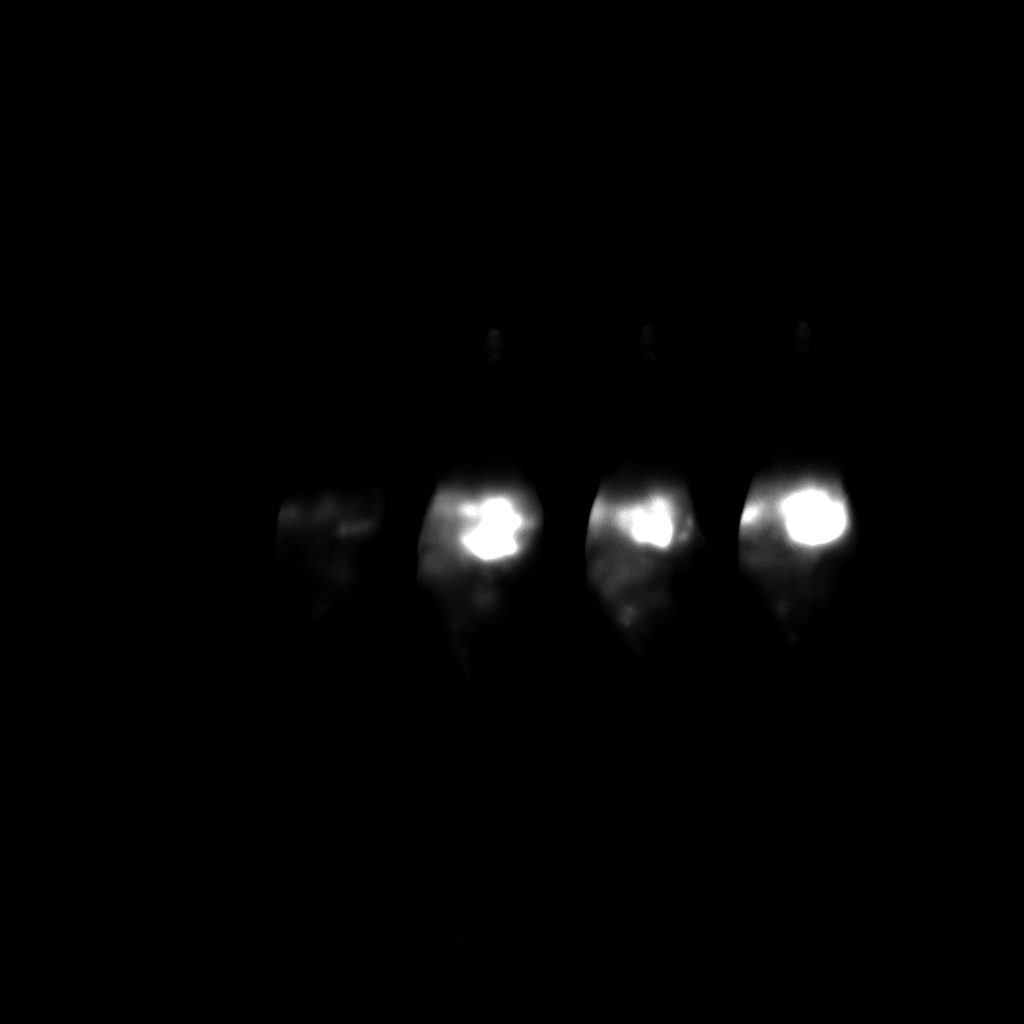

Supplement: Supplementary file 5 — Source data Fig. 4 [file 44320_2026_210_MOESM5_ESM.zip › Fig 4_YW637_hydrodynamic transfection representative images/ACh_185-189_pre-3_8__lumin.png]

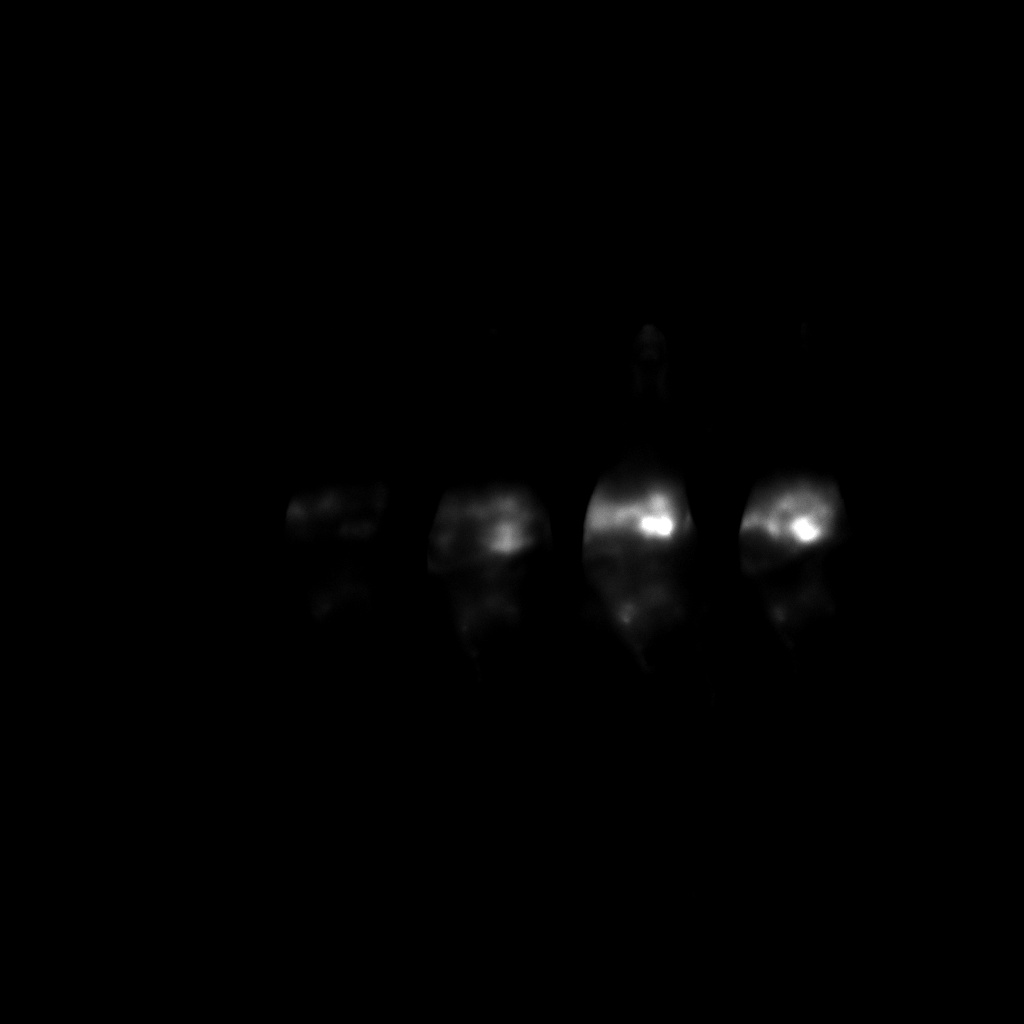

Supplement: Supplementary file 5 — Source data Fig. 4 [file 44320_2026_210_MOESM5_ESM.zip › Fig 4_YW637_hydrodynamic transfection representative images/ACh_185-189_post-2_11__lumin.png]
