# Supplementary material for: Fast analysis and engineering of protein function by microbe-independent deep assembly and screening
Source: Mol Syst Biol. 2026 Apr 23;22(6):1003–34. doi: 10.1038/s44320-026-00210-z (PMC13230610; doi:10.1038/s44320-026-00210-z)
Supplement: Supplementary file 9 — Expanded View Figures [file 44320_2026_210_MOESM9_ESM.pdf]

Expanded View Figures

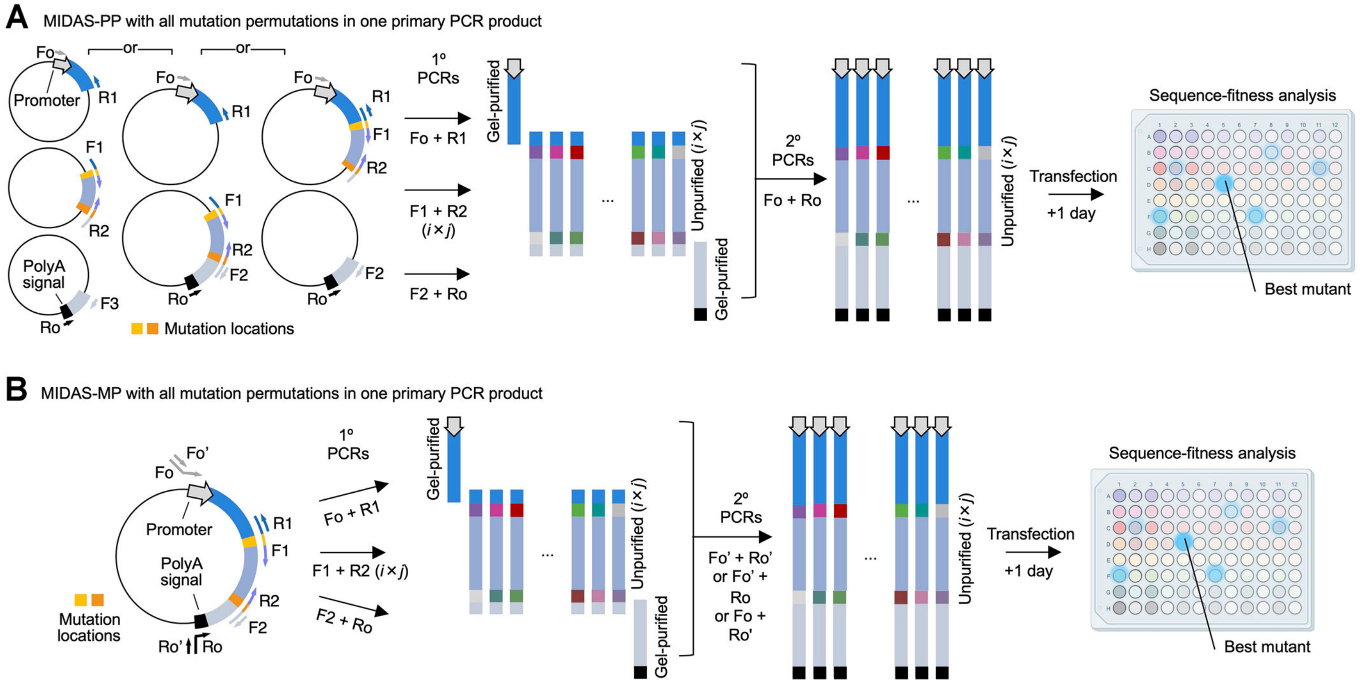

Figure EV1. Alternative schemes for MIDAS-PP and MIDAS-MP.

(**A**) MIDAS-PP with all mutation permutations in one primary PCR product. (**B**) MIDAS-MP with all mutation permutations in one primary PCR product. These schemes require a larger number of primary PCRs (i × j) to cover all permutations at two locations compared to the scheme shown in Fig. 1. However, they may be preferable for robotic handling, as combinatorially mixing oligos to set up i × j primary PCRs and then proceeding one-by-one to the same number of secondary PCRs could be more efficient than combinatorially diluting completed primary PCR products to set up i × j secondary PCRs. Source data are available online for this figure.

© The Author(s)

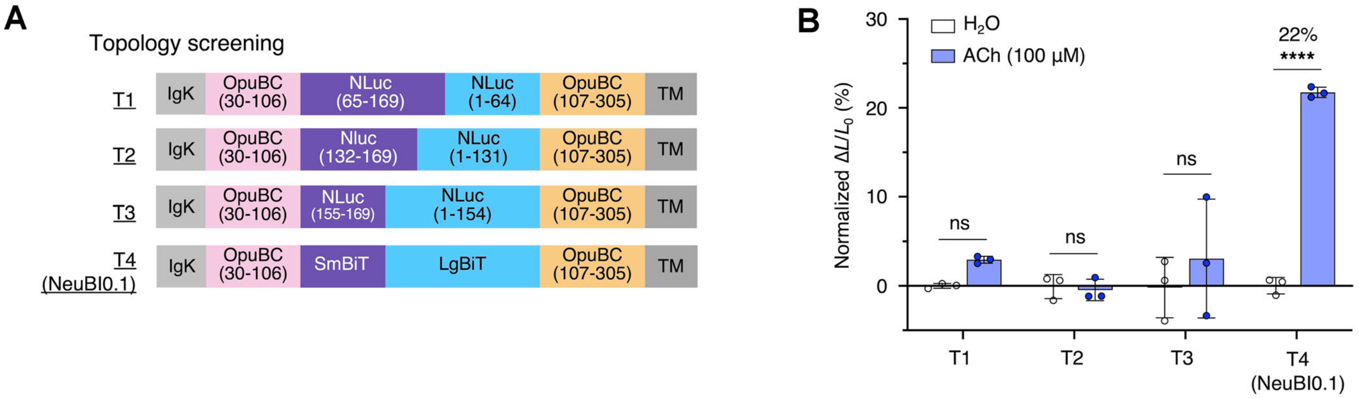

Figure EV2. Screening insertions of circularly permuted forms of NanoLuc into OpuBC.

(**A**) Topologies T1–T4 testing various circular permutation sites of NanoLuc. (**B**) Luminescent signal change of T1–T4 upon the treatment of ACh (100 µM), normalized to the H_2O control. $p = 4.29 \times 10^{-6}$. *$p < 0.05$; **$p < 0.01$; ***$p < 0.001$; ****$p < 0.0001$, by unpaired two-tailed Student's *t*-test compared to the H_2O control. Data were presented as mean ± SD. Three technical replicates. Source data are available online for this figure.

 © The Author(s)

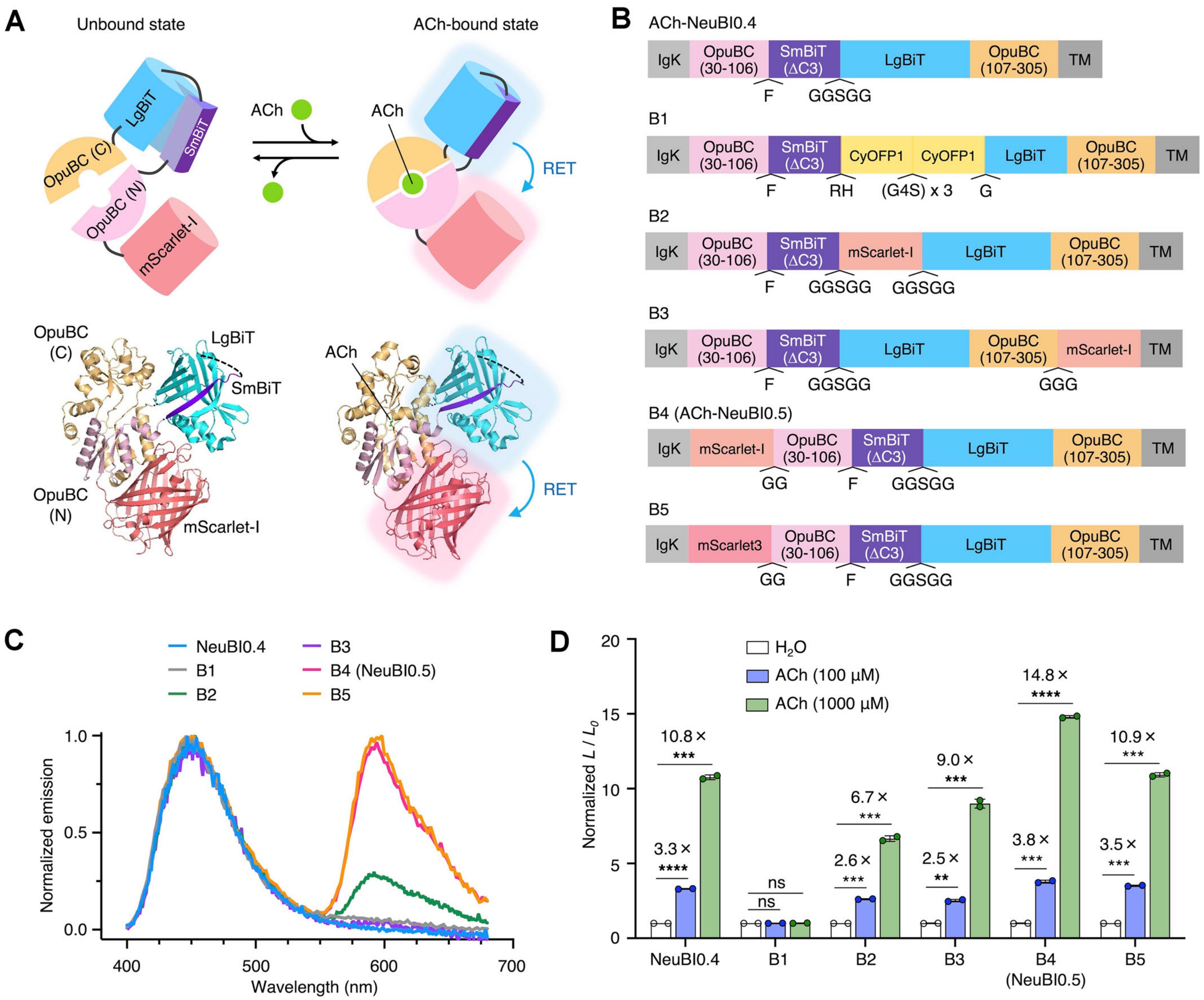

Figure EV3. Engineering of ACh-NeuBI0.5.

(**A**) Proposed mechanism of ACh-NeuBI0.5. Top, cartoon scheme of ACh-NeuBI0.5. Bottom, the structure model of ACh-NeuBI0.5, modified from PDB files 6URU (OpuBC), 5IBO (NanoLuc), and 5LK4 (mScarlet). ACh binding to OpuBC leads to an increase in the luminescence of cpNanoLuc and red light emission from mScarlet-I via resonance energy transfer (RET). (**B**) Topologies of constructs B1–B5 for red-shifting spectra. (**C**) Spectra of B1–B5, measured in the presence of ACh (1000 μM). (**D**) Fold of signal increase of B1–B5 in response to 100 μM ACh or 1000 μM ACh, normalized to the H_2O control. NeuBI0.4: 100 μM: $p = 4.12 \times 10^{-5}$, 1000 μM: $p = 1.31 \times 10^{-4}$, B1: 100 μM: $p = 0.252$, 1000 μM: $p = 0.418$, B2: 100 μM: $p = 1.77 \times 10^{-4}$, 1000 μM: $p = 5.53 \times 10^{-4}$, B3: 100 μM: $p = 1.37 \times 10^{-3}$, 1000 μM: $p = 7.04 \times 10^{-4}$, B4: 100 μM: $p = 5.56 \times 10^{-4}$, 1000 μM: $p = 2 \times 10^{-5}$, B5: 100 μM: $p = 1.23 \times 10^{-4}$, 1000 μM: $p = 1.05 \times 10^{-4}$, ns not significant; *$p < 0.05$; **$p < 0.01$; ***$p < 0.001$; ****$p < 0.0001$, by unpaired two-tailed Student's *t*-test compared to the H_2O control. Data were presented as mean ± SD. Three technical replicates. Source data are available online for this figure.

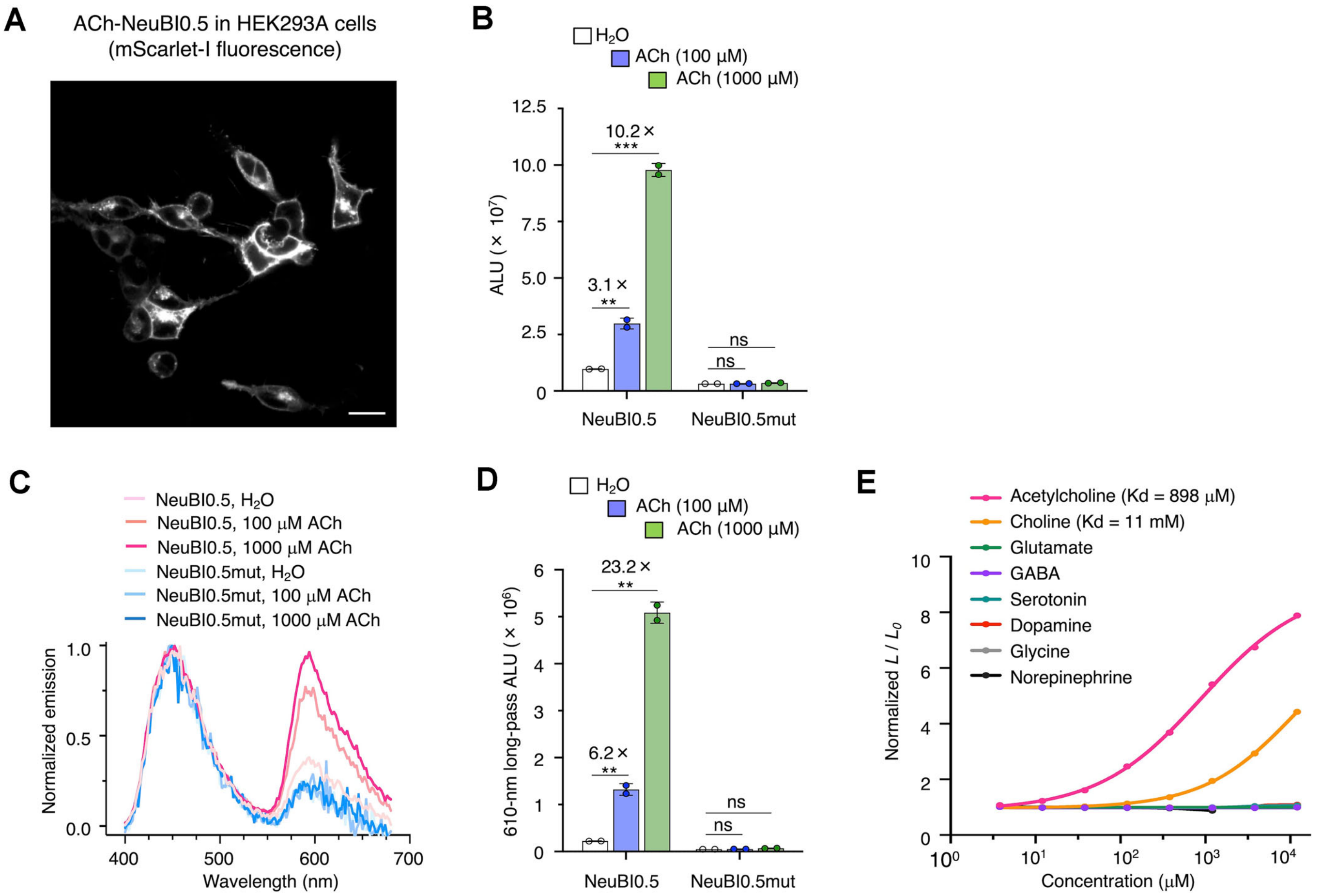

Figure EV4. Characterization of ACh-NeuBI0.5.

(**A**) Confocal microscopy images of mScarlet-I fluorescence of ACh-NeuBI0.5 in HEK293A cells. Scale bar, 20 μm. (**B**) Luminescence of ACh-NeuBI0.5 and ACh-NeuBI0.5mut in 0, 100, or 1000 μM ACh without filter. ALU, arbitrary luminescence units. NeuBI0.5: 100 μM: $p = 7 \times 10^{-3}$, 1000 μM: $p = 5.34 \times 10^{-4}$, ns not significant; $^{*}p < 0.05$; $^{**}p < 0.01$; $^{***}p < 0.001$; $^{****}p < 0.0001$, by unpaired two-tailed Student's *t*-test compared to the H_2O control. Data were presented as mean ± SD. Two technical replicates. (**C**) Spectra of ACh-NeuBI0.5 and ACh-NeuBI0.5mut in 0, 100, or 1000 μM ACh. (**D**) Luminescence of ACh-NeuBI0.5 and ACh-NeuBI0.5mut as in (**B**) but with a 610 nm longpass filter. NeuBI0.5: 100 μM: $p = 6.3 \times 10^{-3}$, 1000 μM: $p = 1.08 \times 10^{-3}$, Data were presented as mean ± SD. Two technical replicates. (**e**) Fold signal increase of ACh-NeuBI0.5 in response to a panel of neurotransmitters at different concentrations on HEK293A cells. NE norepinephrine. Data were presented as mean ± SEM. Three technical replicates. Source data are available online for this figure.

 © The Author(s)

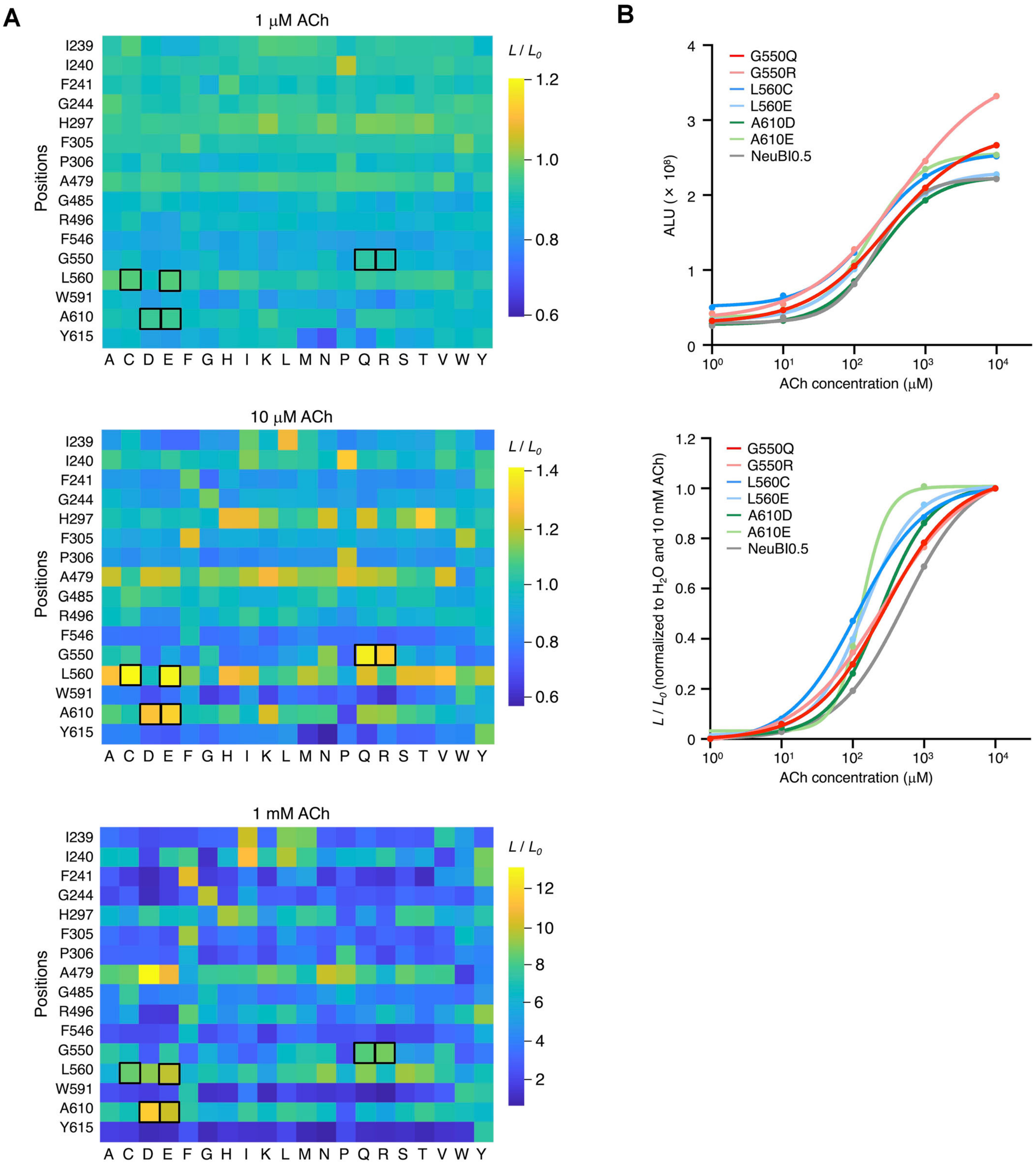

Figure EV5. MIDAS results from saturation mutagenesis at 16 sites.

(**A**) Heatmap showing MIDAS results from saturation mutagenesis at 16 sites in HEK293A cells, tested at 1 μM (top), 10 μM (middle), and 1 mM (bottom) ACh. Boxed cells indicate the top-performing mutants selected for further characterization. (**B**) Dose-response curves of the top mutants from the 16-site saturation mutagenesis in HEK293A cells, raw signals (top), or responses normalized to H_2O and 10 mM ACh (bottom). All curves were fitted using the "nonlinear regression (log(inhibitor) vs. response – variable slope)" model in Prism. Data were presented as mean. Three technical replicates. Source data are available online for this figure.

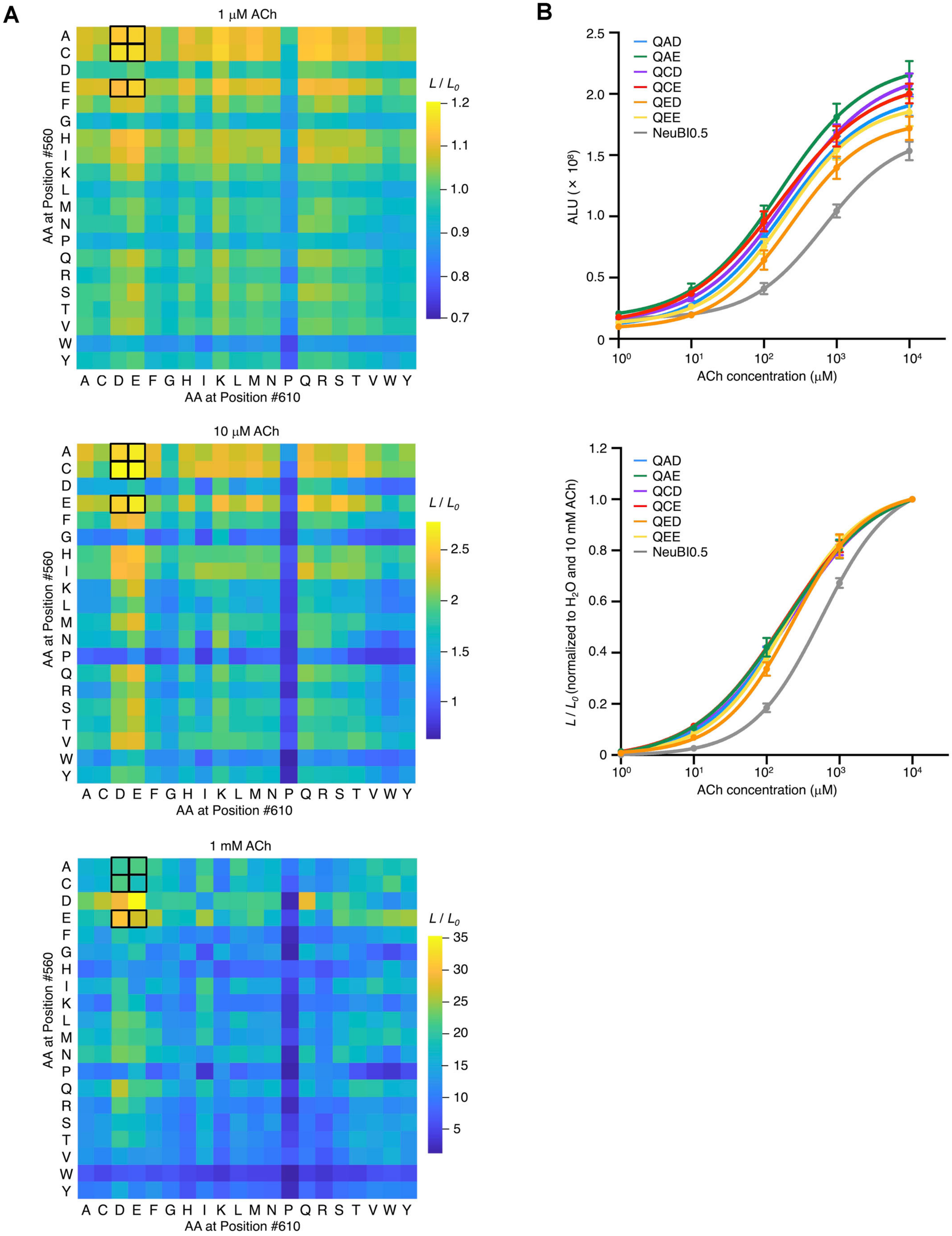
A
1 μM ACh
10 μM ACh
1 mM ACh
AA at Position #560
AA at Position #610
L / L0
B
QAD
QAE
QCD
QCE
QED
QEE
NeuBI0.5
ALU (× 10^8)
ACh concentration (μM)
L / L0 (normalized to H2O and 10 mM ACh)

 © The Author(s)

◀ **Figure EV6. MIDAS results from 20 × 20 combinatorial mutagenesis at positions 560 and 610.**

(**A**) Heatmap showing MIDAS results from 20 × 20 combinatorial mutagenesis at positions 560 and 610 in HEK293A cells, tested at 1 μM (top), 10 μM (middle), and 1 mM (bottom) ACh. Boxed cells indicate the top-performing mutants selected for further characterization. (**B**) Dose-response curves of the top mutants from the 20 × 20 combinatorial mutagenesis in HEK293A cells, raw signals (top), or responses normalized to H_2O and 10 mM ACh (bottom). All curves were fitted using the "nonlinear regression (log(inhibitor) vs. response – variable slope)" model in Prism. Data were presented as mean ± SD. Three technical replicates.

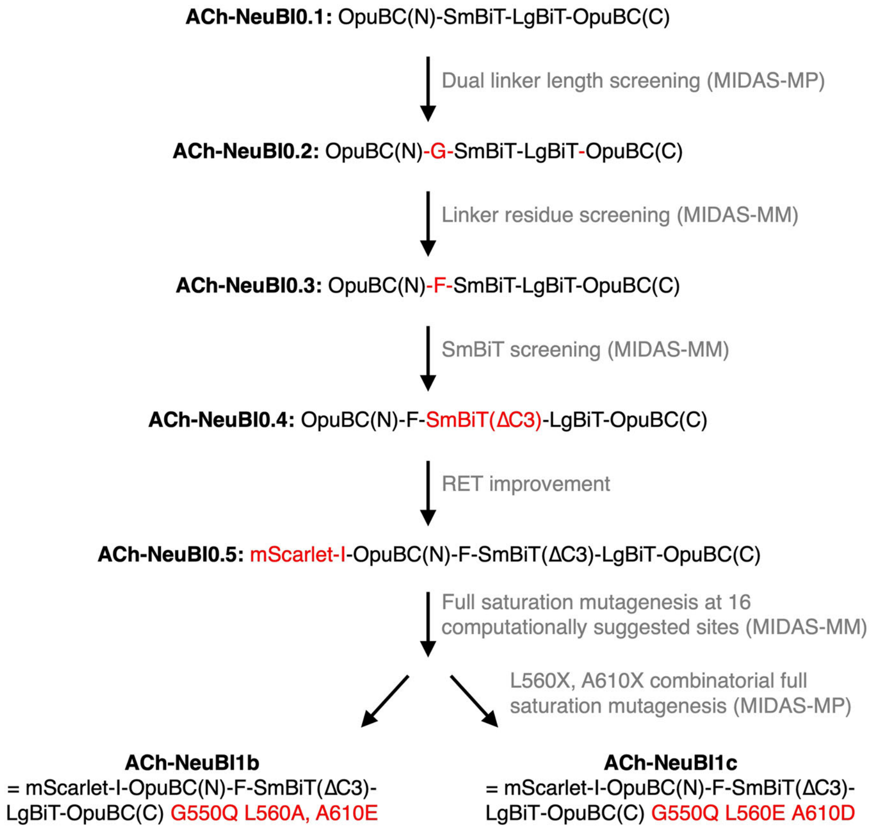

Figure EV7. Evolutionary history of ACh-NeuBIs.

The flowchart depicts the six engineering steps leading to ACh-NeuBI1b and ACh-NeuBI1c from ACh-NeuBI0.1. Molecular changes selected after each step are shown in red. Source data are available online for this figure.

 © The Author(s)

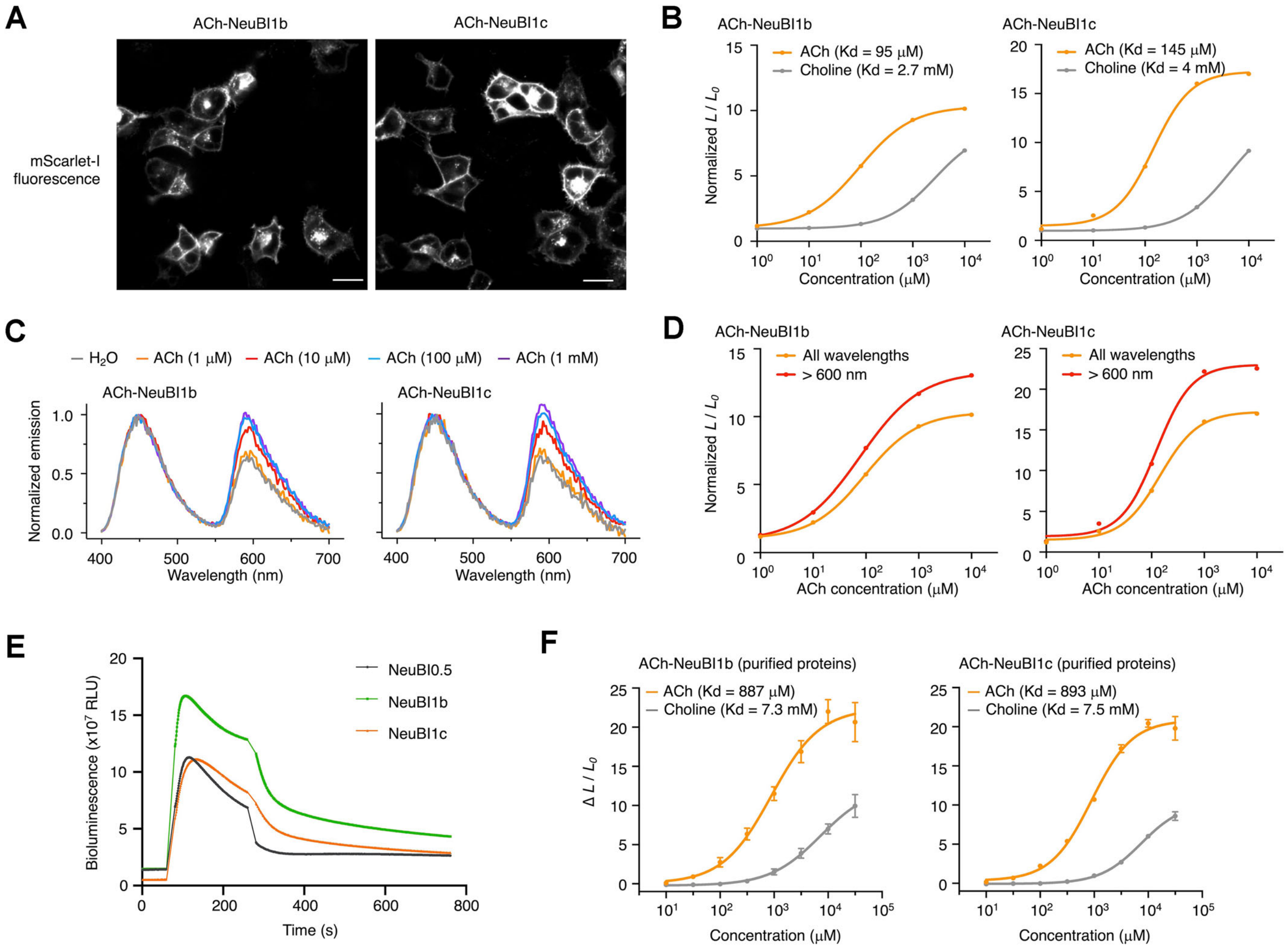

Figure EV8. Characterization of ACh-NeuBI1b and NeuBI1c.

(**A**) Confocal microscopy images of mScarlet-I fluorescence of NeuBI1b (left) and NeuBI1c (right) in HEK293A cells. Scale bar, 20 μm. (**B**) Fold of signal increase of NeuBI1b (left) and NeuBI1c (right) in response to ACh or choline at different concentrations in HEK293A cells, normalized to the H_2O control. (**C**) Spectra of NeuBI1b (left) and NeuBI1c (right), measured without ACh, or with ACh at various concentrations. (**D**) Fold of signal increase of NeuBI1b (left) and NeuBI1c (right) in response to ACh at different concentrations in HEK293A cells without a filter (orange line) or with a 610 nm longpass filter (red line). (**E**) Reversibility of ACh NeuBIs. HEK293A cells expressing individual ACh NeuBIs were imaged under sequential bioluminescence recording. Substrate-containing medium was applied during the initial 0–60 s. A 10 mM ACh solution was then added from 60–240 s to activate the sensors. After 240 s, the medium was replaced with a substrate-containing solution lacking ACh to assess signal reversibility. (**F**) Dose-dependent response of purified NeuBI1b (left) and NeuBI1c (right) proteins to ACh treatment. (**B**, **D–F**) Curves fitted by the "nonlinear regression (log(inhibitor) vs. response – variable slope)" model in Prism. Data were presented as mean ± SD. Three technical replicates. Source data are available online for this figure.

© The Author(s)

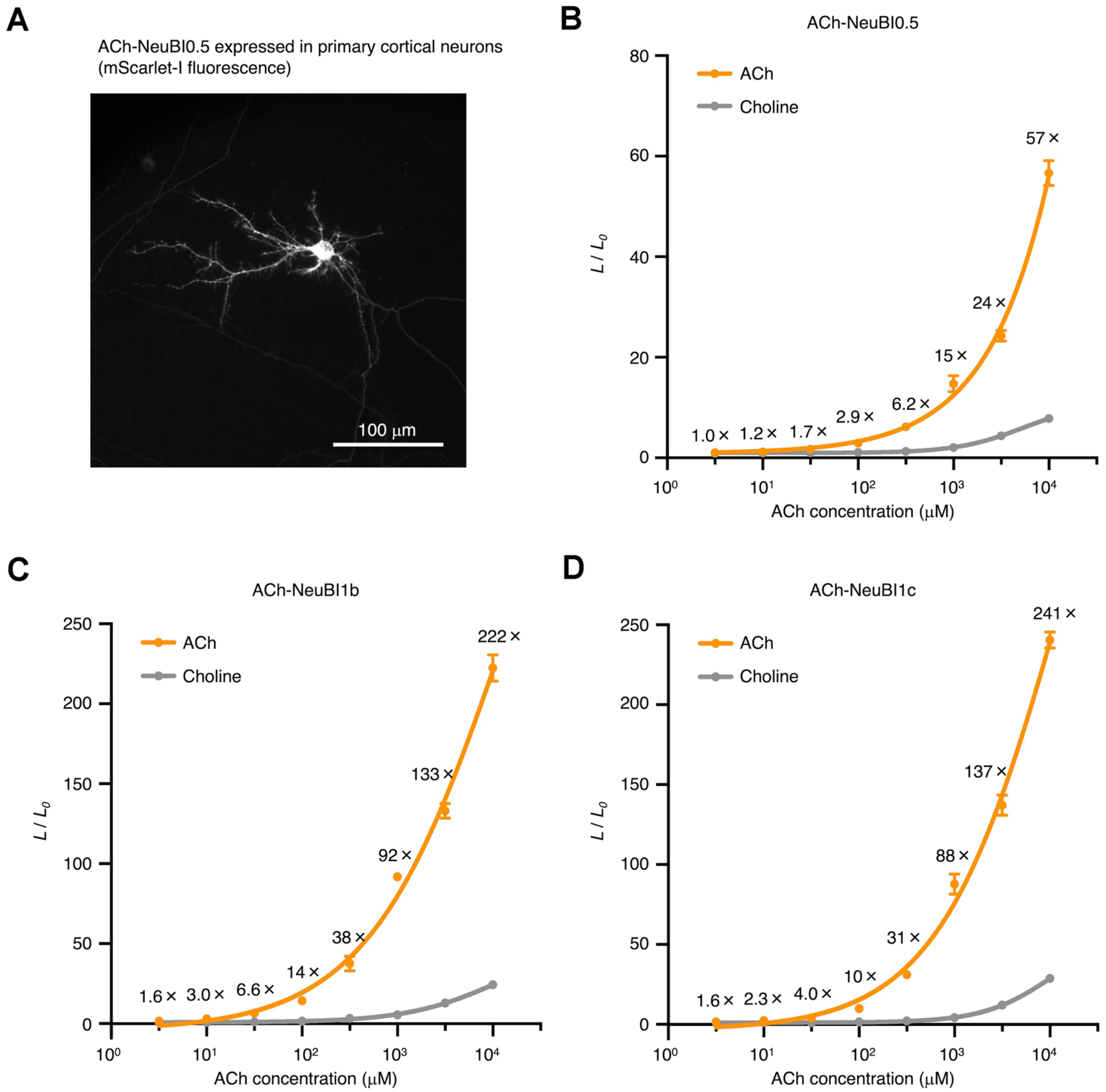

Figure EV9. Characterization of ACh-NeuBIs in cultured neurons.

(**A**) mScarlet-I fluorescence of ACh-NeuBI0.5 in primary cortical neurons. Scale bar, 100 μm. (**B–D**) Fold of signal increase of NeuBI0.5 (**B**), NeuBI1b (**C**), and NeuBI1c (**D**) in response to ACh or choline at different concentrations in primary cortical neurons. Error bars, SEMs. Curves fitted by the "nonlinear regression (log(inhibitor) vs. response – variable slope)" model in Prism. Three technical replicates. Source data are available online for this figure.

 © The Author(s)

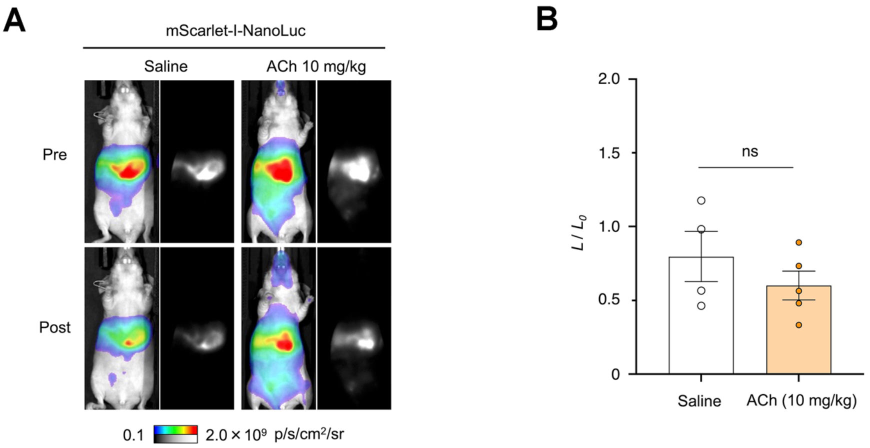

Figure EV10. ACh does not affect NanoLuc's brightness in vivo.

(**A**) Representative bioluminescence images acquired before and after the treatment of saline or ACh (10 mg/kg) in mScarlet-I-NanoLuc-expressing mice. (**B**) Fold of signal increase in response to ACh (10 mg/kg), normalized to the saline control. ns not significant, by unpaired two-tailed Student's *t*-test. Data were presented as mean ± SEM. One biological replicate and four-five technical replicates. Source data are available online for this figure.

© The Author(s)

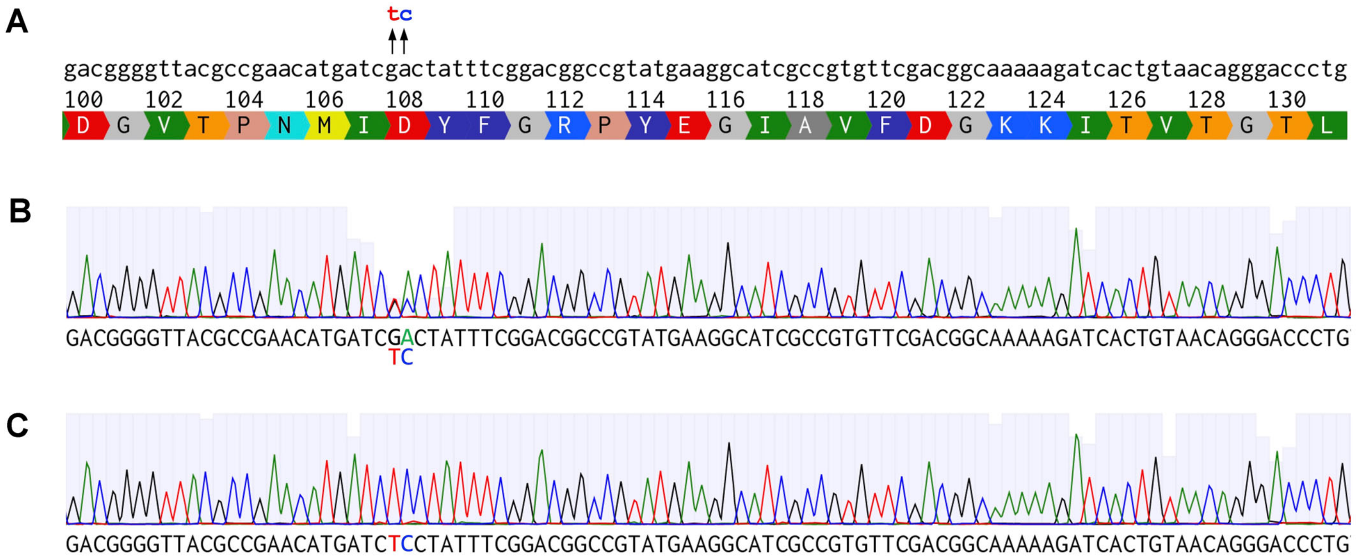

Figure EV11. Modified primer design in MIDAS-MM allows primary PCR dilution into the secondary PCR while avoiding plasmid template re-amplification.

(**A**) DNA sequence and translated protein sequence surrounding two nucleotides targeted for mutation by MIDAS. (**B**) Representative sequencing reaction result of the secondary PCR product using flanking primers that also recognize gene elements in the template used for the mutagenic primary PCR. Primary PCRs were performed to generate a left-side invariant portion and an overlapping right-side mutated portion of a CMV-NanoLuc-BGHpA gene, where CMV is the cytomegalovirus promoter and where BGHpA is the bovine growth hormone gene polyadenylation signal. The forward and reverse primers for the left-side PCR were denoted as F1 and R1 primers, and the forward and reverse primers for the right-side PCR were denoted as F2 and R2 primers, and both primary PCRs used the same template plasmid. A 50-µL secondary PCR was then performed with F1 and R2 primers, and with gel-purified left-side fragment (1 µL of a 1 ng/uL eluate) and 1 µL of unpurified right-side PCR added to supply overlapping templates. When combined with direct secondary PCR transfection into mammalian cells, this method is referred to as MIDAS-PM. A representative Sanger sequencing result is shown with the detected bases underneath. The chromatogram was visualized with the program Benchling (Benchling, Inc.), and bases at the mutated positions were colored to match the chromatogram. (**C**) The same procedure was performed but with two changes: unique sequence extensions were added to the 5′ ends of the F1 and R2 primers used in the primary PCRs, and new F1′ and R2′ primers that primed off the 5′ ends of the F1 and R2 primers, respectively, were used for the secondary PCR. When combined with direct secondary PCR transfection into mammalian cells, this protocol for monotemplated monofocal variant generation and expression is referred to as MIDAS-MM. Sanger sequencing detects only the desired GA to TC mutation.

© The Author(s)
